# β-TCP/S53P4 Scaffolds Obtained by Gel Casting: Synthesis, Properties, and Biomedical Applications

**DOI:** 10.3390/bioengineering10050597

**Published:** 2023-05-16

**Authors:** Suelen Simões Amaral, Beatriz Samara de Sousa Lima, Sarah Oliveira Marco Avelino, Bruno Roberto Spirandeli, Tiago Moreira Bastos Campos, Gilmar Patrocínio Thim, Eliandra de Sousa Trichês, Renata Falchete do Prado, Luana Marotta Reis de Vasconcellos

**Affiliations:** 1 Institute of Science and Technology, São Paulo State University (UNESP), 777 Eng. Francisco José Longo Avenue, São José dos Campos 12245-000, SP, Brazil; suelen.amaral@unesp.br (S.S.A.); bs.lima@unesp.br (B.S.d.S.L.); sarah.avelino@unesp.br (S.O.M.A.); renata.prado@unesp.br (R.F.d.P.); 2 Bioceramics Laboratory, Federal University of São Paulo (UNIFESP), 330 Talim St, São José dos Campos 12231-280, SP, Brazil; bruno.spirandeli@alumni.usp.br (B.R.S.); eliandra.sousa@unifesp.br (E.d.S.T.); 3 Division of Fundamental Sciences, Technological Institute of Aeronautics (ITA), 50 Mal. Eduardo Gomes Plaza, São José dos Campos 12228-900, SP, Brazil; moreiratiago22@gmail.com (T.M.B.C.); gilmar@ita.br (G.P.T.)

**Keywords:** bioceramic scaffolds, β-TCP, bioactive glass S53P4, sol–gel, osteogenesis, bone neoformation

## Abstract

The objective of this study was to investigate the osteogenic and antimicrobial effect of bioactive glass S53P4 incorporated into β-tricalcium phosphate (β-TCP) scaffolds in vitro and the bone neoformation in vivo. β-TCP and β-TCP/S53P4 scaffolds were prepared by the gel casting method. Samples were morphologically and physically characterized through X-ray diffraction (XRD) and scanning electron microscope (SEM). In vitro tests were performed using MG63 cells. American Type Culture Collection reference strains were used to determine the scaffold’s antimicrobial potential. Defects were created in the tibia of New Zealand rabbits and filled with experimental scaffolds. The incorporation of S53P4 bioglass promotes significant changes in the crystalline phases formed and in the morphology of the surface of the scaffolds. The β-TCP/S53P4 scaffolds did not demonstrate an in vitro cytotoxic effect, presented similar alkaline phosphatase activity, and induced a significantly higher protein amount when compared to β-TCP. The expression of Itg β1 in the β-TCP scaffold was higher than in the β-TCP/S53P4, and there was higher expression of Col-1 in the β-TCP/S53P4 group. Higher bone formation and antimicrobial activity were observed in the β-TCP/S53P4 group. The results confirm the osteogenic capacity of β-TCP ceramics and suggest that, after bioactive glass S53P4 incorporation, it can prevent microbial infections, demonstrating to be an excellent biomaterial for application in bone tissue engineering.

## 1. Introduction

Calcium phosphate ceramics are the most used and effective synthetic biomaterial bone substitutes [1]. β-TCP is a ceramic biomaterial used both in dentistry and medicine [2,3]. As bone substitutes, β-TCP scaffolds are attractive because they have biocompatibility, adequate absorption, and bone neoformation [4,5]. Moreover, β-TCP is considered osteoconductive and osteoinductive [6]. Previous studies have reported successful in vivo surgical implantation of biomaterials produced with β-TCP ceramic [7,8].

In 1969, another ceramic biomaterial was developed by Larry Hench, 45S5 bioglass (Bioglass^®^), considered a promising material due to its chemical bonding to bone tissue [9]. The osteoinductive property of bioglass has been described since it presents the ability to dissolve into soluble silica and calcium ions, which promotes the stimulation of osteogenic cells to produce bone matrix [10]. However, other formulations have been investigated to assure the osteogenic and antimicrobial properties of bioglasses [11,12]. Bioactive glass S53P4 (53% SiO_2_, 23% Na_2_O, 20% CaO, and 4% P_2_O_5_, wt%) is considered a biocompatible and osteoconductive bone substitute. Furthermore, it has antibacterial, osteo-stimulating, and angiogenic properties [13].

Studies suggest that the incorporation of bioactive glass into β-TCP scaffolds increases its mechanical strength and bioactivity [14,15], as well as the osteo-stimulating and osteoconductive capacity of bioglass [16,17], positively interfering in the performance of the β-TCP scaffold. Thus, combining the β-TCP and bioactive glass S53P4 is ideal to produce scaffolds for bone tissue engineering, as bone substitutes, in the repair and replacement of damaged tissue [8].

Waselau et al. [18] evaluated the effect of S53P4 and β-TCP on osteogenic differentiation of human adipose stem cells (hASCs), separately, presenting a granular shape. ALP staining indicated osteogenic differentiation of hASCs in bioglass and β-TCP groups. Alves et al. [19] produced and characterized 3D-printed β-TCP/S53P4 scaffolds. According to the authors, the scaffolds were effective in terms of antibacterial activity and cell viability.

Scaffolds are expected to provide support for cell proliferation and differentiation, diffusion of cellular nutrients, and exert mechanical and biological influences on cells [20]. To provide an adequate structure in scaffolds, porosity, pore size, and interconnected pores are important characteristics for adequate osteogenesis [21,22]. The optimization of these parameters can be achieved in the fabrication of porous ceramic scaffolds using the gel-casting method [23].

In this study, β-TCP scaffolds were incorporated with bioactive glass S53P4 to investigate in vitro osteoblastic activity, differentiation, and gene expression, related to osteogenesis, through Real-Time Quantitative Reverse Transcription Polymerase Chain Reaction (RT-PCR), as well as to evaluate in vivo bone neoformation. The antibacterial activity of these scaffolds was also verified against ATCC strains of *P. aeruginosa*, *S. aureus*, and *C. albicans*.

## 2. Materials and Methods

### 2.1. Fabrication of β-TCP Scaffolds by Gel Casting Method and Incorporation of Bioactive Glass S53P4 by Sol–Gel

The β-TCP powder was obtained by solid-state reaction of anhydrous bibasic calcium phosphate CaHPO_4_ and calcium carbonate CaCO_3_ (both from Synth—Diadema, Brazil) according to a procedure described previously [15]. The powder was characterized by X-ray diffraction (XRD) (Rigaku-Ultima IV, Tokyo, Japan, 10–60°, 0.02°, 10 mm/s) to verify the crystalline phases formed in the β-TCP. β-TCP scaffolds were obtained using the gel casting method. Briefly, a ceramic suspension of 30% in weight of β-TCP was prepared with an aqueous solution including 15% of organic monomers hydroxymethylacrylamide, methacrylamide, and methylenebisacrylamide in a 3:3:1 M ratio. The production of ceramic foam occurred by adding a foaming agent (Lutensol ON-110, BASF, Ludwigshafen, Germany), followed by mechanical agitation in a ball mill for 20 min. The foam was conditioned into cylindrical molds of polyvinyl chloride (7 × 2 mm). The molds were maintained 24 h at room temperature (23 °C) and 24 h at 70 °C. The scaffolds were kept in an oven at 100 °C for 24 h. The gelled foams were then demolded and then sintered at 1200 °C/2 h.

The synthesis of bioglass sol was carried out by adding the precursors NaNO_3_ (0.098% mol), Ca(NO_3_)_2_·4H_2_O (0.110% mol), and (NH_4_)H_2_PO_4_ (0.010% mol) in a beaker containing a solution of silicic acid (H_4_SiO_4_ 0.185% mol). At the end of the additions, a transparent bioglass sol was obtained, which was used to impregnate the β-TCP scaffolds.

The bioactive glass incorporation procedure was performed by immersion of the scaffold in 200 mL of the sol–gel bioglass solution in a vacuum chamber at −1 Bar pressure. After 30 min under vacuum, the scaffolds were removed from the solution and dried for 24 h at 100 °C. A detailed procedure for the manufacture of scaffolds by the gel casting method and incorporation of bioactive glass into β-TCP scaffolds has been previously described [15].

### 2.2. Characterization of β-TCP and β-TCP/S53P4 Scaffolds

#### 2.2.1. X-ray Diffraction (XRD) Analysis

The determination of the crystalline phases present in the scaffolds was carried out in the macerated β-TCP and β-TCP/S53P4 scaffolds. For this purpose, a model X’pert Powder diffractometer (X’Pert PRO MPD 3060 PANalytical, Almelo, The Netherlands) was used. Standards from 20° to 40° on the 2θ axis were collected, with a scan step of 10.1600 s, step size of 0.0170°, and CuKα radiation.

#### 2.2.2. Morphological and Physical Characterization of β-TCP and β-TCP/S53P4 Scaffolds

Morphological analysis of the surface of the scaffolds produced by the gel casting method was performed using images obtained by low-vacuum scanning electron microscope using thermal emission electron optics SEM (FEI, Inspect S50 model, Brno, Czech Republic). SEM images and *Image J software* bundled with 64-bit Java 8. (National Institutes of Health, Bethesda, MD, USA) were used to determine average pore sizes.

The geometric porosity of the β-TCP and β-TCP/S53P4 scaffolds was determined according to Equations (1) and (2). Where “*d*” is the scaffold geometric density and “*d_Theoretical_*” corresponds to the theoretical density of β-TCP (3.07 g/cm^3^), “*m_Scaffold_*” is the measured mass in (g) and “*V_Scaffold_*” is the calculated volume in (cm^3^) of the scaffolds.

(1) $$P\left(\%\right)=\left[1-\left(\frac{d_{Scaffolds}}{d_{teoric}}\right)\right]\times 100$$

(2) $$d_{scaffolds}=\left(\frac{m_{Scaffolds}}{V_{Scaffolds}}\right)$$

### 2.3. Biological Characterization of β-TCP and β-TCP/S53P4 Scaffolds

#### 2.3.1. In Vitro Cell Culture

Cellular studies were conducted using human MG63 cells (APABCAM, Rio de Janeiro, Brazil), a human osteoblast cell line. Cells were grown in Dulbecco’s Modified Eagle Medium (DMEM, Cultilab, São Paulo, Brazil), supplemented with 10% fetal bovine serum (FBS, Cultilab, São Paulo, Brazil) and gentamicin (10 mg/mL) (Gibco™, Paisly, UK). Incubated at a temperature of 37 °C in a humid atmosphere containing 5% CO_2_, when confluence was reached by occupying more than 80%, the cells were ready for seeding. Cells were separated by trypsinization (0.25% Trypsin-EDTA Gibco™, Paisly, UK) and centrifuged (Labnet Centrifuge—HERMLE Z 300K, Winooski, VT, USA), then resuspended with culture medium. Before seeding, scaffolds were placed in Petri dishes and sterilized under UV irradiation for 30 min. Cells were calculated using a hemocytometer (Prolab, São Paulo, Brazil) and were pipetted into each scaffold in 48-well plates (Sarstedt^®^, Numbrecht, Germany) at a cell density of 15 × 10^4^ cells for the tests described below, except for the test of gene expression. Replicates were performed according to ISO 10993-5, with three repetitions of the experiments and five scaffolds for each test in each repetition.

##### Cell Morphology

After 5 days of culture, two scaffolds of each material were fixed with Karnovisky’s solution (4% paraformaldehyde (Neon, Suzano, Brazil), 2.5% glutaraldehyde, and 0.1 M sodium cacodylate buffer (both Dinâmica São Paulo, Brazil, pH 7.4) for 2 h; then, the scaffolds were dehydrated with ethanol. The scaffolds were coated with a thin layer of gold in a sputter-coating system (Q150R ES, Quorum London, UK) for scanning electron microscope (SEM) analysis (EVO MA10 Zeiss, Jena, Germany).

Other two scaffolds were fixed with 4% paraformaldehyde in 0.1M Dulbecco phosphate buffer (PBS Gibco, New York, NY, USA), pH 7.2, and washed with PBS. Then, the cells were processed by direct fluorescence to verify their cytoskeleton using 0.5% Triton X-100 (Sigma-Aldrich, St. Louis, MO, USA), followed by blocking unspecific background with 5% skimmed milk in PBS. A solution of Alexa Fluor 488 phalloidin (1:200, Molecular Probes Invitrogen, Waltham, MA, USA) in PBS was incubated in a humidified environment at room temperature. Finally, the cell nuclei were stained with 300nM 4′,6-diamidino-2-phenylindole dihydrochloride (Fluorshiel^TM^ with Dapi Sigma-Aldrich, St. Louis, MO, USA). Next, samples were examined under epifluorescence using a Leica DMLB light microscope (Leica, Bensheim, Germany) fitted with a Leica DC 300F digital camera.

##### Cell Viability

After 3 and 7 days, the culture medium was removed from each well. MTT solution (0.05% of 3-(4,5-dimethylthiazol-2-yl)-2,5-diphenyltetrazolium bromide— M5655 Sigma-Aldrich, St. Louis, MO, USA) in culture medium was added to the cultured samples and incubated for 4 h to form formazan crystals that were dissolved by DMSO (Dimethylsulfoxide, Neon, Suzano, Brazil). The absorbance at 570 nm was measured in a microplate reader (EL808IU Biotek Instruments, Winooski, VT, USA). The optical densities obtained were converted into percentages in relation to the number of live cells in the β-TCP, representing 100% of cell viability.

##### Total Protein Content

The total protein content was determined through Lowry’s modified method [24]. At 7 and 14 days of culture, proteins were extracted with 0.1% sodium dodecyl sulfate (Sigma-Aldrich, St. Louis, MO, USA). Cell lysates were added to Lowry solution (Sigma-Aldrich, St. Louis, MO, USA) and Folin–Ciocalteau (Sigma-Aldrich, St. Louis, MO, USA) reagents. The absorbance corresponding to the proteins was measured at 690 nm in a microplate reader (EL808IU Biotek Instruments, Winooski, VT, USA).

##### Alkaline Phosphatase (ALP) Assay

After 7 and 14 days of cell culture, the same cell lysates above were incubated following the instructions of the commercial kit manufacturer (Labtest Diagnostica, Lagoa Santa, Brazil) for ALP activity determination. In ALP assay, thymophthalein is released by hydrolysis of the substrate thymophthalein monophosphate. Absorbance was measured in a microplate reader (EL808IU Biotek Instruments, Winooski, VT, USA) at 590 nm.

##### Alizarin Red Staining

The staining of mineralization nodules was evaluated after 14 days of cell culture using 2% alizarin red (Sigma-Aldrich, St. Louis, MO, USA), which stains calcium-rich areas. The cultures received Hank’s solution (H6136 Sigma-Aldrich, St. Louis, MO, USA) and 2% alizarin S red dye. The formation of mineralization nodules was observed under an optical microscope (Axio Observer A1, Carl Zeiss, Germany).

##### RNA Extraction and Real-Time Quantitative Reverse Transcription PCR (qRT-PCR)

Further, 1 × 10^5^ MG63 cells were seeded in scaffolds, and the qRT-PCR test was used to assess the expression of specific osteogenic genes, COL-1, TGF-Β1, ITG-β1, M-CSF, OSN, BGLAP, OSP, PGE_2_, RUNX2, and housekeeping gene β-actin (Table 1). After 7 days in culture, the total RNA was extracted using Trizol (Ambion^®^, Life Technologies Corporation, Van Allen Way, Carlsbad, CA, USA) according to the manufacturer’s instructions. Further, cDNA was synthesized by reverse transcription reactions following the manufacturer’s instructions of the commercial SuperScript III kit, First-Strand Synthesis Supermix (Invitrogen Life Technologies Corporation-Van Allen Way, Carlsbad, CA, USA), and cDNA was used for qRT-PCR with the Step One Plus Time PCR thermocycler detection system (Thermofisher Scientfic Inc., Waltham, MA, USA) using the Platinum SYBR Green qPCR SuperMix-UDG system (Invitrogen Life Technologies Corporation-Van Allen Way, Carlsbad, CA, USA) and specific primers according to the manufacturer’s instructions. The relative quantification was calculated for each gene by the comparative method of ΔΔCt [25].

#### 2.3.2. Metabolic Activity of Microorganisms to Assess the Antimicrobial Effect of Scaffolds

Reference strains (ATCC—American Type Culture Collection) of *C. albicans* (ATCC 18804), *P. aeruginosa* (ATCC 15442), and *S. aureus* (ATCC 6538) from the Laboratory of Microbiology and Immunology of the Institute of Science and Technology of UNESP, São José dos Campos Campus, Brazil, which were stored in a freezer at −80 °C, were used for this study. The colonies were diluted in sterile saline solution (0.9% NaCl) and standardized to 10^7^ cells/mL in a spectrophotometer (B582, Micronal, São Paulo, Brazil) according to the wavelength and optical density of each microorganism.

The scaffolds were distributed in each well of the 48-well plate (Kasvi, Paraná, Brazil), with n = 05 per experimental group. For microbial growth, the specific standardized cells’ suspensions were added over to the scaffold on the culture plate and fed with BHI broth. The culture medium was renewed within 24 h. A growth control group of each microorganism was carried out without the presence of the scaffolds.

The plates were incubated in an oven at 37 °C for 48 h. Subsequently, 0.5 mg/mL MTT solution (Sigma-Aldrich, St. Louis, MO, USA) was added to the broth. After 1h of incubation, under protection from light, this solution was removed and DMSO (Sigma-Aldrich, St. Louis, MO, USA) was added, followed by absorbance reading (Biotek, model ELx808cse Winooski, VT, USA) at 570 nm.

#### 2.3.3. Animals and Implantation Procedure

Five male albino New Zealand white rabbits were used, weighing about 4.0 kg, at 5 months of age. They were purchased from the central vivarium of São Paulo State University. The animals were placed in individual cages and received standard commercial food and distilled water ad libitum. They were acclimatized to the housing conditions over four weeks at 20–24 °C and 50–70% relative humidity with a 12 h light/dark cycle.

Each animal received two scaffolds in each tibia, divided according to the manufacturing material: (a) β-TCP and (b) β-TCP/S53P4 scaffolds.

All experiments were performed following the Animal Ethics Committee (CEUA, Protocol 02/2020) of the Institute of Science and Technology of the Campus of São José dos Campos/UNESP and were carried out under the ethical principles adopted by the Brazilian National Animal Care Ethical Council (CONCEA). All surgical procedures were performed under general anesthesia in a pain-free state. The research followed all the recommendations of the “Animal Research: Reporting in vivo Experiments” (ARRIVE) guidelines [26].

The animals were weighed and anesthetized with a 2% xylazine hydrochloride solution (2 mg/100 mL)—(Anasedan^®^—Vetbrands, Jacareí, Brazil) and 1.16 g/10 mL ketamine hydrochloride (Dopalen^®^—Vetbrands, Jacareí, Brazil). After anesthesia, shaving, and antisepsis, with iodinated alcohol solution, the surgery was started in the medial region of the rabbit’s tibia. The cortical bone of the tibia was exposed, and surgical pockets were formed. Two perforations of 4 mm in diameter were performed in each tibia, totaling 4 perforations per animal, with the left tibias receiving β-TCP scaffolds and the right tibias receiving β-TCP/S53P4 scaffolds. They received one oral dose of 5mg/kg of tramadol hydrochloride (Eurofarma, São Paulo, Brazil) to prevent post-operative pain.

The animals were inspected daily for clinical signs of complications or adverse reactions to arrest any suffering to animals. They were monitored until the euthanasia period. The animals were euthanized with an overdose of deep general anesthesia at 21 days of implantation. For histological observations, implanted scaffolds were fixed in buffered formalin and decalcified using 20% formic acid.

##### Histological Procedure

After demineralization, specimens were submitted to routine histological processing, embedding paraffin, and sectioned in 5 µm slices. Sections were stained with hematoxylin and eosin (HE) and observed in a light microscope Zeiss Axiophot 2 (Carl Zeiss, Oberköchen, Germany).

##### Bone Tissue Formation Evaluation by Area Measurement

The bone tissue formation area was measured below the pre-existing cortical bone in the region of the bone defect. Digital images were obtained at an original magnification of 5X using a light microscope Zeiss Axiophot 2 associated with Axiocam MRC 5 (Zeiss Oberköchen, Germany). Thereafter, 100 areas of each material were analyzed using Image J software bundled with 64-bit Java 8. (National Institutes of Health, Bethesda, MD, USA).

### 2.4. Statistical Analysis

Obtained data were tested by the Kolmogorov–Smirnov test and passed the normality test. Independent samples *t*-test was used for cell viability, total protein content, alkaline phosphatase assay, qRT-PCR, and bone tissue formation evaluation. The microbial metabolic activity was analyzed through the one-way analysis of variance (ANOVA) and Tukey multiple comparisons analysis. A *p*-value < 0.05 was considered statistically significant. All data are presented as mean and standard deviation. Graphs and statistics were performed through GraphPad Prism^®^ software version 8.

## 3. Results and Discussion

**Figure 1:**
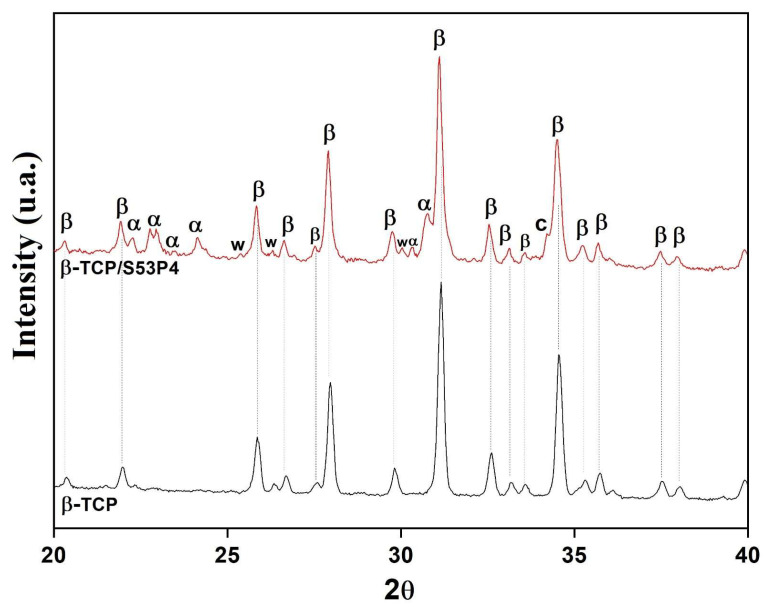
XRD patterns of β-TCP and β-TCP/S53P4 scaffolds. β: β-TCP; α: α-TCP; c: Na_2_Ca_2_Si_3_O_9_; w: CaSiO_3_.

Figure 1 shows the XRD diffractograms of β-TCP and β-TCP/S53P4 scaffolds. Only the β-TCP crystalline phase (β-Ca_3_(PO_4_)_2_, JCPDS 00-09-0169) was verified in the β-TCP scaffold. The β-TCP/S53P4 scaffold presented, in addition to the β-TCP phase, the crystalline phases α-TCP (α-Ca_3_(PO_4_)_2_, JCPDS 00-09-0348), combeite (Na_2_Ca_2_Si_3_O_9_, JCPDS 00-022-1455), and wollastonite (CaSiO_3_, JCPDS 00-027-0088). The formation of the α-TCP phase has been observed in Si-doped β-TCP [27,28,29]. This has been attributed to the action of incorporated silicon: the Si^4+^ enters the network occupying positions of the tetrahedral P^5+^ in the β-TCP structure and stabilizes the α-TCP phase at temperatures below theoretical [27,28,29]. On the other hand, calcium and sodium–calcium silicates are phases commonly obtained in the crystallization of bioglasses in the system SiO_2_-Na_2_O-CaO-P_2_O_5_ [30,31,32]. The crystallization of these phases favors the bioactivity of the composite since, while β-TCP has a low capacity for apatite mineralization in vivo and in vitro, the α-TCP phase and silicates are highly bioactive phases capable of inducing rapid mineralization in vivo [30,33,34,35].

**Figure 2:**
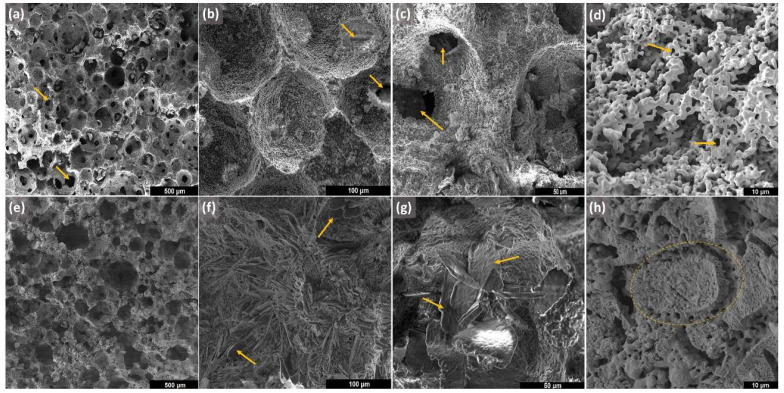
SEM micrographs of β-TCP and β-TCP/S53P4 scaffolds. β-TCP: (**a**) 100×, (**b**) 1000×, (**c**) 2000× (**a**–**c**, yellow arrows indicate openings of interconnected macropores), (**d**) 5000× (**b**–**d**, yellow arrows indicate micropores); β-TCP/S53P4: (**e**) 100×, (**f**) 1000×, (**g**) 2000× (**f**,**g**, yellow arrows indicate plates with several microns, (**h**) 5000× (dashed circle highlights formed silicate).

Figure 2a–h shows SEM micrographs of β-TCP (Figure 2a–d) and β-TCP/S53P4 (Figure 2e–h) scaffolds. The scaffolds presented morphology characteristic of the foam gel casting method, with a cellular structure consisting of macropores interconnected by several openings, as indicated by the arrows in Figure 2a–c, and micropores with smaller sizes at 1 μm on the struts, indicated by the arrows in Figure 2d. After bioactive glass incorporation, β-TCP/S53P4 scaffolds showed a significant change in strut surface morphology. The formation of several structures having the form of plates with several microns in length was observed, as indicated by the arrows in Figure 2f,g. These structures have a typical morphology of combeites [36,37] and were associated with sodium–calcium silicate formed by the crystallization of the incorporated bioglass, observed in XRD analysis. The observation of β-TCP/S53P4 scaffold struts under higher magnifications (5000×) also showed regions covered with layers of the formed silicates, as illustrated by the dashed region in the micrograph of Figure 2h. Similar results were found by Spirandeli et al. [15] when bioactive glass 45S5 was incorporated into β-TCP scaffolds by sol–gel.

The β-TCP scaffold presented a porosity of 76%, and β-TCP/S53P4 scaffolds showed a porosity of 79%, both ±1.0%, and mean pore sizes of 195 μm and 148 μm, respectively. The incorporation of S53P4 on the scaffolds did not significantly affect the porosity and pore size, which are important parameters for osteogenesis; they are related to cell recruitment, adhesion, cell proliferation, as well as nutrient permeability [32]. As human cancellous bone exhibits a total porosity ranging from 30% to 90%, a scaffold presenting porosity within this range is suitable for bone regeneration [21].

There are three levels of porosity in bone, which are inserted hierarchically one inside another, associated with the vascular, lacunar–canalicular, and collagen–apatite porosities. Their typical dimensions are described as 50 μm, 100 nm, and 1 nm, respectively [38]. Both scaffolds presented mean pore sizes compatible with vascular bone porosity, and those pores being interconnected is crucial to osteogenesis inside the scaffolds.

Hoover et al. [39] developed highly porous silver-doped β-TCP scaffolds (77% and 79%) and concluded that porosity increased osteoconduction. Seidenstuecker et al., 2017 [40] characterized pure β-TCP scaffolds combined with bioactive glass, produced through 3D printing, with porosity between 63% and 71%. They concluded that the biomaterial of BG/β-TCP with greater porosity promoted a high concentration of MG63 living cells. There are conflicting reports in the literature regarding the ideal pore size. Roy et al., 2003 [41] demonstrated adequate osseointegration in rabbit calvaria defects with poly (L-lactic acid-co-glycolic acid) and β-TCP scaffold in pores with a mean diameter between 125 and 150 μm. Karageorgiou and Kaplan, 2005 [21] reported that pores > 300 μm are recommended as they promote the adequate formation of new bone and capillaries. However, for Murphy and O’Brien, 2010 [42], the ideal pore size for bone formation is between 85 and 120 μm.

**Figure 3:**
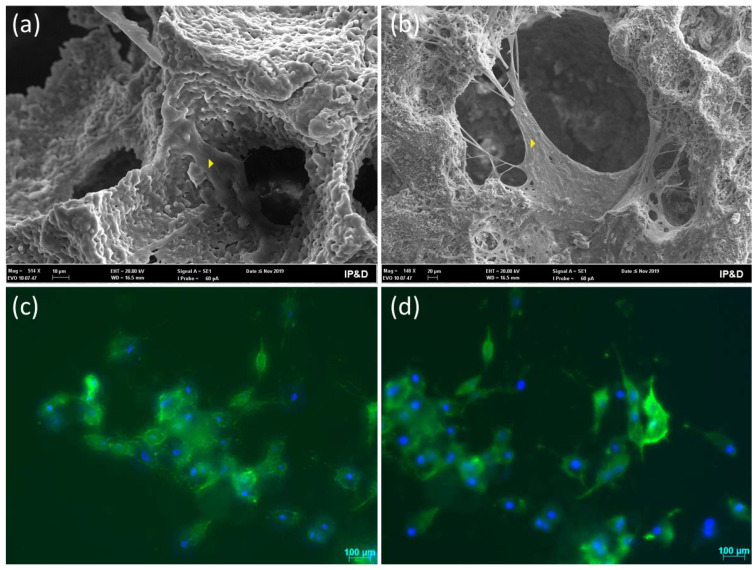
Cell morphology: SEM micrographs of cells adhered to the surface (540× magnifications). (**a**) The yellow arrow points to a cell on the surface of the β-TCP scaffold. The cell presents a smooth surface, and the cytoplasmic boundaries show the formation of processes (pseudopodia). (**b**) The yellow arrow points to a cell on the surface of the β-TCP/S53P4 scaffold. The cell adhered to the entrance of a pore and sends out web-like projections. (**c**) Direct fluorescence of cells adhered to the surface of β-TCP scaffolds, and (**d**) β-TCP/S53P4 scaffold showing adherent cells with a stellate polygonal morphology and multidirectional spreading.

Figure 3 shows SEM micrographs of adherent cells on the β-TCP and β-TCP/S53P4 scaffolds’ surfaces in Figure 3a,b and direct fluorescence of cells in Figure 3c,d. The cells that adhered to the scaffolds exhibited elongated morphology, evidencing the interaction with scaffolds independent of material. In both fluorescence figures, cell nuclei are shown in blue, stained by DAPI, while cytoskeletons are shown in green, stained by Alexa Fluor 488.

**Figure 4:**
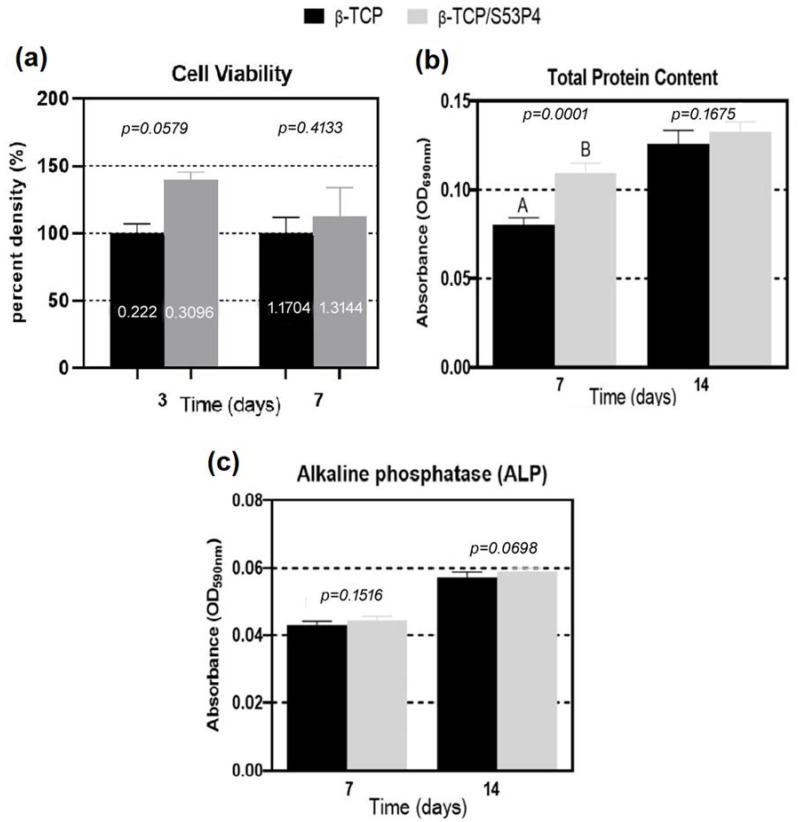
Graph of the mean (± standard deviation) of values obtained in the (**a**) cell viability of β-TCP and β-TCP/S53P4 scaffolds after 3 and 7 days (*p* > 0.05). (**b**) Total protein test (PT) and (**c**) ALP graph showing alkaline phosphatase activity at 7 and 14 days of cell culture. Different letters indicate statistical differences (*p* < 0.05).

The cell viability of MG63 cells in β-TCP and β-TCP/S53P4 scaffolds was measured by MTT test. Figure 4a shows that the β-TCP/S53P4 scaffolds did not induce a toxic effect on the MG63 osteoblast cell line compared to β-TCP independently of the period. An increase in cell viability was observed in the group incorporated with bioactive glass. At 3 days, the viability was 139%, and, at 7 days, it was 112% but without a significant difference when compared to β-TCP (*p* > 0.05). Tao et al., 2020 [43] demonstrated that scaffolds constituted of calcium phosphate did not exhibit cell toxicity. In this research, after the manufacture of β-TCP scaffolds, bioglass was incorporated into the scaffolds via immersion in a sol–gel solution. There is not yet a consensus in the literature regarding the mechanism of dissociation of bioglass ions in a conditioned medium. It is described that, on the surface of the bioglass, the microenvironment is transformed through the liberation of ions that cause an increase in pH in the culture medium, which, according to the authors, can be harmful to cells [44]. In this study, bioglass incorporation did not change cell viability, suggesting the absence of cytotoxicity on osteoblasts. Previous results support that structures functionalized with Ca^2+^ ions promote the adhesion, proliferation, and differentiation of MG63 cells [45]. Bioactive glass S53P4 is composed of ions that provide beneficial biological properties that stimulate osteogenesis [16]. Waselau et al., 2012 [18] analyzed the cell proliferation of human adipose stem cells at periods of 1, 7, and 14 days, measuring the amount of DNA in the samples. They observed a higher cell proliferation rate in S53P4 bioglass granules compared to β-TCP.

The incorporation of S53P4 into β-TCP scaffolds had a favorable impact on cell cultures, improved cell viability, increased the total protein content, and did not induce a reduction in alkaline phosphatase activity. ALP is an essential marker in osteoblast differentiation [46,47]. The phosphatase concentration increases as osteoblast differentiation occurs, resulting in bone matrix formation and its calcification [48]. Figure 4b shows that β-TCP/S53P4 scaffolds expressed higher total protein production compared to β-TCP scaffolds in both periods but with a statistical difference at 7 days (*p* < 0.05).

It appears that, once the difference in cell viability between the two groups decays with time (illustrated by the MTT assay values at 3 and 7 days), the difference in total protein content between these groups also decays.

Contact with bioactive glass scaffolds for longer periods induces greater differentiation in bone cells (reflected by the increasing value of alkaline phosphatase activity levels at 14 days when compared to 7 days). Cells committed to differentiation have less proliferative activity. The protein-synthesizing activity, exhibited by the total intracellular protein content of the osteoblasts, could reflect the proliferation ability of the osteoblasts to some extent [49], justifying the absence of significant difference, at 14 days between the two groups, in the protein levels.

The results of the ALP assay (Figure 4c) show no significant difference (*p* > 0.05) between β-TCP and β-TCP/S53P4 scaffolds regardless of the period of 7 or 14 days of culture. There was an increase in the expression of total protein content and ALP activity because of culture time. These results are similar to those described by Pandey et al., 2013 [50], in which ceramic materials, based on zirconia and alumina, were exposed to MG63 cells during 8 and 16 days of culture. Zhang et al., 2003 [51] also observed higher ALP activity in the later cell culture period in the group containing calcium phosphate glass in chitosan composite scaffolds.

**Figure 5:**
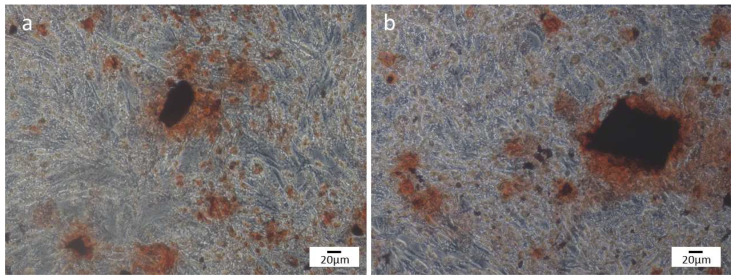
Alizarin red staining of mineralized nodule formation in vitro at 14 days: (**a**) βTCP and (**b**) β-TCP/S53P4 scaffolds.

The formation of mineralized nodules was observed in both scaffolds. Figure 5a,b illustrates the formation of mineralization nodules after 14 days of cell culture. In Figure 5b, a larger mineralization nodule is observed in the β-TCP/S53P4 scaffold when compared to the β-TCP, suggesting that the incorporation of bioactive glass S53P4 to β-TCP scaffolds may favor the mineralization of the matrix produced by MG63 cells. Previous studies have shown increased mineralization in bioglass materials, such as bioglass scaffolds containing PCL-based graphene nanopowder [52]. Gong et al., 2017 [53] demonstrated greater formation of mineralization nodules by MG63 cells cultivated in 58S nanometric bioglass when compared to 45S5. Paramita et al., 2021 [54] reported the formation of mineralization nodules by rats’ mesenchymal stem cells in contact with nano-bioglass ceramics with zinc (Zn–nBGC), cultivated with and without the osteogenic medium.

**Figure 6:**
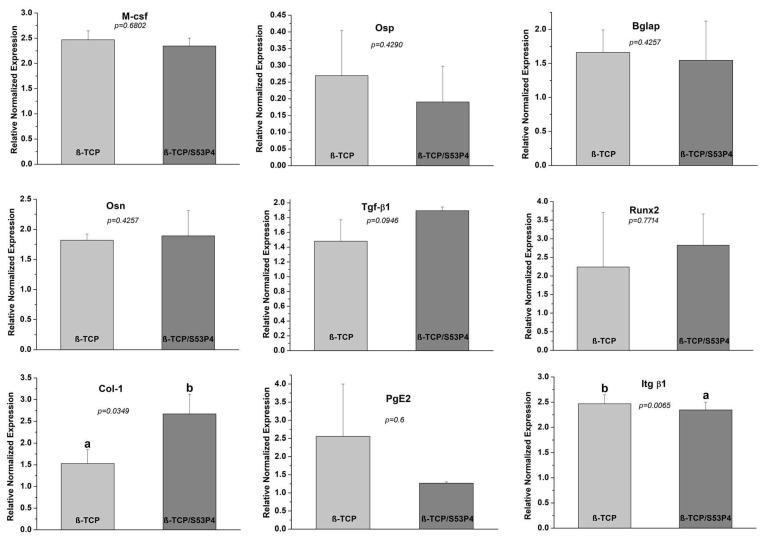
M-CSF, Osp, Bglap, Osn, Tgf- β1, Runx2, Col-1, PgE_2,_ and Itg β 1 expression at day 7. Values are normalized by the β-Actin housekeeping gene. The Student’s *t*-test demonstrated differences in the expression levels of Col-1 and Itg β1. *P*-values are observed in the figure. β-TCP promoted lower expression of collagen I and higher expression of integrin β1 than β-TPC/S53P4 scaffolds. Different letters indicate statistical differences (*p* < 0.05).

Figure 6 summarizes the molecular results. Cells cultivated in contact with β-TCP and β-TCP/S53P4 scaffolds presented no significant differences (*p* > 0.05) in the expression of M-CSF, BGLAP, OSP, PGE2, and RUNX2 genes.

M-CSF is a cytokine that regulates the proliferation and differentiation of osteoclast, monocyte, and macrophage precursors [55,56] and also acts as an anti-inflammatory and immunosuppressive agent for implantable biomaterials [47]. The M-CSF gene was expressed in β-TCP and β-TCP/S53P4 scaffolds without significant difference (*p* > 0.05). Regarding non-collagenous proteins, Osp is a multifunctional adhesive glycoprotein containing arginine–glycine–aspartate acid (RGD), which acts as an integrin ligand [57]. Furthermore, it is an adhesion molecule, which can bind to multiple cell surface receptors involved in cell–cell and cell–matrix interactions [58]. Bglap is synthesized only by osteoblasts; it is the most abundant non-collagen protein in the bone extracellular matrix that binds to calcium ions. Increased Bglap expression is related to osteoblast differentiation; it is a late marker of mineralization [59,60]. Osn is a protein expressed in mineralized and non-mineralized tissue, secreted by osteoblasts during bone formation. It is also responsible for cellular interactions and cell binding to calcium and Col-1 [61] and can induce angiogenesis and neovascularization [62]. Although without significant difference (*p* > 0.05), β-TCP/S53P4 scaffold induced greater expression of the Osn gene than the β-TCP scaffold. Additionally, the growth factors of Tgf-β can regulate Osn [63]. Our results demonstrate the same pattern of Osn and Tgf-β1 expression on scaffolds. Although without significant difference (*p* > 0.05), the β-TCP/S53P4 scaffold induced greater expression of Tgf-β1 genes than the β-TCP scaffold.

Runx2 is a transcription factor expressed by osteoblasts that is fundamental in osteoblast differentiation [64]. It is a very common marker in osteoblast differentiation protocols; it is expressed during the initial phase by pre-osteoblasts of the cell cycle until the beginning of matrix maturation [65]. Ke et al., 2019 [66] analyzed, in vitro, the expression of the Runx2 gene in a pre-osteoblast cell line in contact with pure β-TCP disc and β-TCP associated with strontium oxide, silica, magnesia, and zinc oxide. They observed Runx2 upregulation in the β-TCP scaffold on the third day, and, on the ninth day, its downregulation occurred. Thus, they concluded that the downregulation of Runx2 demonstrated its inhibitory role at the end of the osteoblastic differentiation stage. In this research, the evaluation was at 7 days and the Runx2 gene presented higher mean expression in scaffolds containing bioactive bioglass but without a significant difference between scaffolds.

Col-1 is the most abundant protein in the bone matrix, and its main function is structural [67]. It has been shown that the Osterix gene (a Runx2 modulate gene) can upregulate Col1a1 expression [68]. Initially, in bone formation, osteoblasts produce osteoid matrix through collagen deposition, and, during the final stage, the rate of collagen synthesis decreases [69]. In this study, there was higher expression of Col-1 in β-TCP/S53P4 when compared to the β-TCP scaffold. This result suggests the positive influence of β-TCP/S53P4 in osteoblastic activity due to the higher expression of Col-1.

Prostaglandins are multifunctional regulators of bone resorption and formation related to skeletal metabolism, bone inflammation, and consolidation. They are abundantly expressed in bone tissue because of COX-2 stimulation [70]. In this study, the PgE2 was more highly expressed in the β-TCP group when compared with β-TCP/S53P4 scaffolds but without significant difference (*p* > 0.05).

Itg β1 is a cell surface molecule involved in many different biological processes: migration, growth, differentiation, and apoptosis [71]. Itg β1 is essential for osteoclast bone resorption and osteoblast function [72]. Itg β1 expression was upregulated in cells in contact with β-TCP scaffolds (*p* < 0.05).

**Figure 7:**
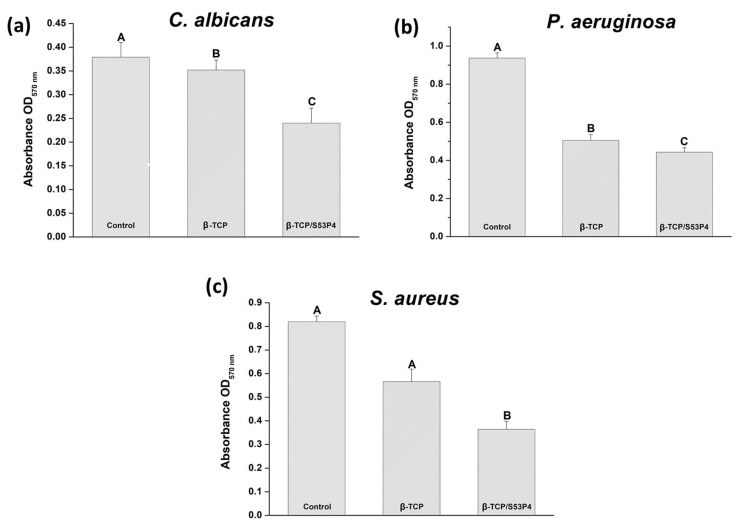
Graph of mean values (± standard deviation) of optical density obtained from biofilm formations of (**a**) *C. albicans*; (**b**) *P. aeruginosa*; (**c**) *S. aureus* after the MTT test on the different materials. Different letters indicate significant differences from the Tukey multiple comparisons test (*p* < 0.05).

According to the results (Figure 7), bioactive glass S53P4 incorporated into β-TCP scaffolds had an inhibitory effect on all tested pathogens. After 48 h of incubation, there was a significant reduction in *C. albicans* and *P. aeruginosa* metabolic activity in contact with the β-TCP/S53P4 scaffold when compared to the other groups (*p* < 0.05) (Figure 7a), the results from the β-TCP scaffold also being lower than (*p* > 0.05) the control (Figure 7a).

The β-TCP/S53P4 scaffold demonstrated antimicrobial activity against the tested strain of *S. aureus*, with a significant difference (*p* < 0.05) between the β-TCP scaffolds and the control group (Figure 7c).

The antimicrobial effect of bioactive glass S53P4 occurs due to the increase in pH, creating an alkaline environment through the release of ions from the bioglass, simultaneously increasing the osmotic pressure and causing damage to the bacterial wall [13], and then the effectiveness of the treatment does not depend on combinations with antibiotics [17,73]. The evaluation of the antimicrobial activity of bioactive glass S53P4 granules against different microorganisms was recently demonstrated [74] in bioglass granules (500–800 μm) and powder (<45 μm). An isothermal microcalorimetry test and CFU were performed to test the antimicrobial effect against *S. aureus*, *S. epidermidis*, *Enterococcus faecalis*, *Escherichia coli*, and *C. albicans*. Bioactive glass S53P4 presented an inhibitory effect on all the pathogens tested [74].

Grønseth et al., 2020 [75] also demonstrated the antimicrobial effect on *S. aureus* of S53P4 granules (<45 μm) in direct contact with the bacterial biofilm. Kirchhoff et al., 2020 [76] observed, through SEM, a change in the morphology of the bacterial biofilm of P. aeruginosa, suggesting that this change was a result of the action of the bioglass, which induced a reduction in the density of the extracellular matrix. All these previous studies are in accordance with the results of the present study since they also demonstrated the antimicrobial activity of bioactive glass S53P4.

**Figure 8:**
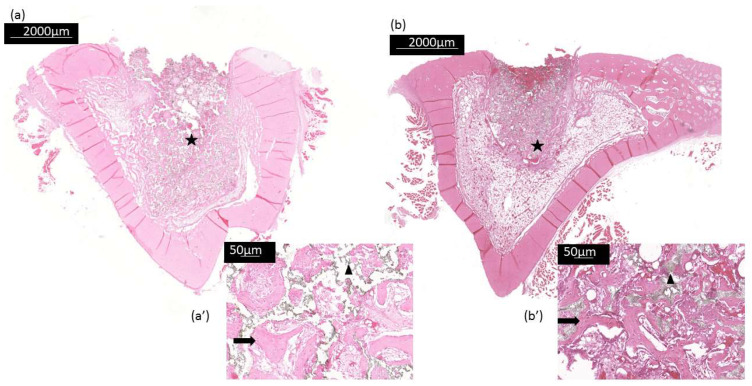
Histological images of bone formation in the (**a**) β-TCP and (**b**) β-TCP/S53P4 scaffold after 21 days of implantation: (**a**,**b**) cross section of the tibia region analyzed for bone formation (black star) (2.5× magnification); (**a′**,**b**′) bone neoformation details (arrow) and material residues (arrowhead) (10× magnification).

This work evaluated the bone neoformation in contact with experimental scaffolds implanted in bone defects in rabbit tibias. There was no tissue infection and no clinical reaction after the surgical implantation of scaffolds in experimental animals. New bone formation was demonstrated by bone trabeculae in the bone defect regardless of the filling material (Figure 8). The bone repair was observed around and inside the pores present in the β-TCP and β-TCP/S53P4 scaffolds (Figure 8a,b). In Figure 8a, in the internal region of the β-TCP scaffold, the osteoid matrix deposit and areas containing scaffold residues were observed. Likewise, in the β-TCP/S53P4 scaffold in Figure 8b, in addition to these aspects, many bone trabeculae were observed. Figure 8a′,b′) shows the direct contact of the scaffolds with the newly formed bone. It is possible to observe close contact between the spaces previously occupied by the scaffold that was degraded and the cells. Inside the newly formed bone trabeculae, there are osteocytes within the lacunae, and osteoblasts are found on the bone surface. Gunn et al., 2013 [77] also identified the formation of trabeculae bone and S53P4 bioglass granule residues within 3 weeks after implantation in rabbits.

**Figure 9:**
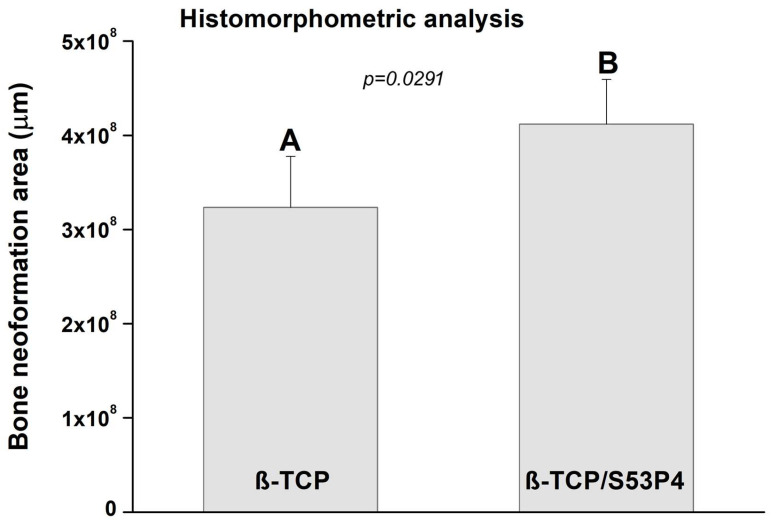
Histomorphometric analysis. Different letters indicate significant differences from Tukey multiple comparisons test (*p* < 0.05).

The results of the histomorphometric analysis are shown in Figure 9. At 21 days, the β-TCP/S53P4 scaffolds induced greater bone formation in the defect area, presenting a significant difference (*p* < 0.05) when compared to β-TCP. Previous studies have proven the osteoconductive properties of this bioglass [78,79].

Signs of bone remodeling in defects filled with β-TCP scaffolds were observed in this study and agree with previous studies [7,8]. Recently, Titsinides et al., 2020 [8] compared several grafts, including β-TCP, derived from human bone, and a bovine xenograft, that were grafted into porcine calvaria. Bone formation was more evident in β-TCP at both 8 and 12 weeks by histomorphometric analysis. However, the results were more relevant at 12 weeks during bone maturation and consolidation.

## 4. Conclusions

The incorporation of sol–gel-produced bioactive glass S53P4 into β-TCP scaffolds promoted significant changes in the morphology and crystalline phases of β-TCP scaffolds. It induced the partial transformation of the β-TCP phase into α-TCP and the crystallization of highly bioactive phases as calcium and sodium–calcium silicates. β-TCP/S53P4 scaffolds did not impair human osteoblasts metabolism or viability; instead, they induced osteogenic markers expression and decreased metabolic activity of *P. aeruginosa*, *S. aureus*, and *C. albicans*. The β-TCP/S53P4 scaffold promoted greater bone neoformation after implantation. Therefore, the β-TCP/S53P4 scaffold proved to be a promising biomaterial for application in the treatment of bone defects and infections.

## Figures and Tables

**Table 1:** Sequence of sense and antisense primers, size of the product in base pairs, and PubMed reference.

Gene	PubMed Reference	Forward Primer	Reverse Primer	Product Length (pb)
Col-1	NM_000088.3	ACAGCCGCTTCACCTACAGC	GTTTTGTATTCAATCACTGTCTTGC	85
Tgf-β1	NM_000660.4	TTTGATGTCACCGGAGTTGTG	GCGAAAGCCCTCAATTTCC	63
Itg β1	NM_002211.3	TTCTTCCTGGACTATTGAAAT	AGAAACTCTCATCATGCTCATT	100
M-csf	NM_172211.3	GAGCTGCTTCACCAAGGATTAT	TCTTGACCTTCTCCAGCAACTG	92
Osn	NM_003118.3	ACTGGCTCAAGAACGTCCTGGT	TCATGGATCTTCTTCACCCGC	97
Bglap	NM_001199662.1	AGCAGAGCGACACCCTAGAC	GGCAGCGAGGTAGTGAAGAG	194
Osp	NM_001251830.1	AGACACATATGATGGCCGAGG	GGCCTTGTATGCACCATTCAA	154
PgE_2_	NM_004878.4	GAAGAAGGCCTTTGCCAA	GGAAGACCAGGAAGTGC	200
Runx2	NM_001015051	GAACTGGGCCCTTTTTCAGA	CACTCTGGCTTTGGGAAGAG	208
β-actin	NM_001101.3	AAACTGGAACGGTGAAGGTG	GTGGACTTGGGAGAGGACTG	206

## Data Availability

Data will be made available on request.
